# MultiOmicsIntegrator: a nextflow pipeline for integrated omics analyses

**DOI:** 10.1093/bioadv/vbae175

**Published:** 2024-11-14

**Authors:** Bianka Alexandra Pasat, Eleftherios Pilalis, Katarzyna Mnich, Afshin Samali, Aristotelis Chatziioannou, Adrienne M Gorman

**Affiliations:** Apoptosis Research Centre, School of Biological and Chemical Sciences, University of Galway, Galway H91TK33, Ireland; Science Foundation Ireland, SFI Centre for Research Training in Genomics Data Science, Galway H91TK33, Ireland; Center of Systems Biology, Biomedical Reseach Foundation of the Academy of Athens, Athens 11527, Greece; e-NIOS Applications PC, Athens 17671, Greece; e-NIOS Applications PC, Athens 17671, Greece; Apoptosis Research Centre, School of Biological and Chemical Sciences, University of Galway, Galway H91TK33, Ireland; CÚRAM, SFI Research Centre for Medical Devices, University of Galway, Galway H91TK33, Ireland; Apoptosis Research Centre, School of Biological and Chemical Sciences, University of Galway, Galway H91TK33, Ireland; CÚRAM, SFI Research Centre for Medical Devices, University of Galway, Galway H91TK33, Ireland; Center of Systems Biology, Biomedical Reseach Foundation of the Academy of Athens, Athens 11527, Greece; e-NIOS Applications PC, Athens 17671, Greece; Apoptosis Research Centre, School of Biological and Chemical Sciences, University of Galway, Galway H91TK33, Ireland; Science Foundation Ireland, SFI Centre for Research Training in Genomics Data Science, Galway H91TK33, Ireland; CÚRAM, SFI Research Centre for Medical Devices, University of Galway, Galway H91TK33, Ireland

## Abstract

**Motivation:**

Analysis of gene and isoform expression levels is becoming critical for the detailed understanding of biochemical mechanisms. In addition, integrating RNA-seq data with other omics data types, such as proteomics and metabolomics, provides a strong approach for consolidating our understanding of biological processes across various organizational tiers, thus promoting the identification of potential therapeutic targets.

**Results:**

We present our pipeline, called MultiOmicsIntegrator (MOI), an inclusive pipeline for comprehensive omics analyses. MOI represents a unified approach that performs in-depth individual analyses of diverse omics. Specifically, exhaustive analysis of RNA-seq data at the level of genes, isoforms of genes, as well as miRNA is offered, coupled with functional annotation and structure prediction of these transcripts. Additionally, proteomics and metabolomics data are supported providing a holistic view of biological systems. Finally, MOI has tools to integrate simultaneously multiple and diverse omics datasets, with both data- and function-driven approaches, fostering a deeper understanding of intricate biological interactions.

**Availability and implementation:**

MOI and ReadTheDocs.

## 1 Introduction

RNA sequencing (RNA-seq) has transformed the resolution and throughput of transcriptome analysis, refining the former to the single nucleotide base, while raising the latter to the order of hundred million reads per sample, enabling a deeper understanding of gene expression and regulation under different conditions. At the same time, its generic and adjustable character allows the measurement of various transcript forms, including gene and isoform levels. Integrating both gene and isoform-level analysis provides the basis for a more nuanced understanding of the molecular mechanisms underlying gene regulation in complex biological systems (Wang *et al.* 2008). Furthermore, the integration of different types of omics data has become increasingly important in biological research, as it enables a more comprehensive understanding of complex biological signaling processes. For example, integrating proteomics with RNA-seq data can reveal post-transcriptional regulation of gene expression, while integrating metabolomics data can provide insights into metabolic pathways involved in the condition under study (Pinu *et al.* 2019).

In this paper, we present our contribution to enhance the analysis of omics datasets by developing MultiOmicsIntegrator (MOI), a nextflow pipeline that focuses on exhaustive RNA-seq analysis, including functional annotation and secondary structure prediction of transcripts, with the additional possibility of integration of diverse omics data, including miRNA, metabolomics and proteomics.

## 2 Methods—implementation

### 2.1 MOI architecture

MOI is a nextflow pipeline (Tommaso *et al.* 2017) with its dependencies stored with Docker (Merkel 2014) that provides an exhaustive, manifold analysis of RNA-seq data and enables integration of other omics data types. Its modular design caters for versatility based on the type of data (RNA-seq, metabolomics, or proteomics), its format (raw files or abundance matrices), and the desired analysis approach (isolated analysis or integration of multiple omics datasets).

Conceptually, MOI is comprised of three major parts that are described below. The most important tools are listed in Supplementary Table S1.

### 2.2 Data pre-processing

**Figure 1.**
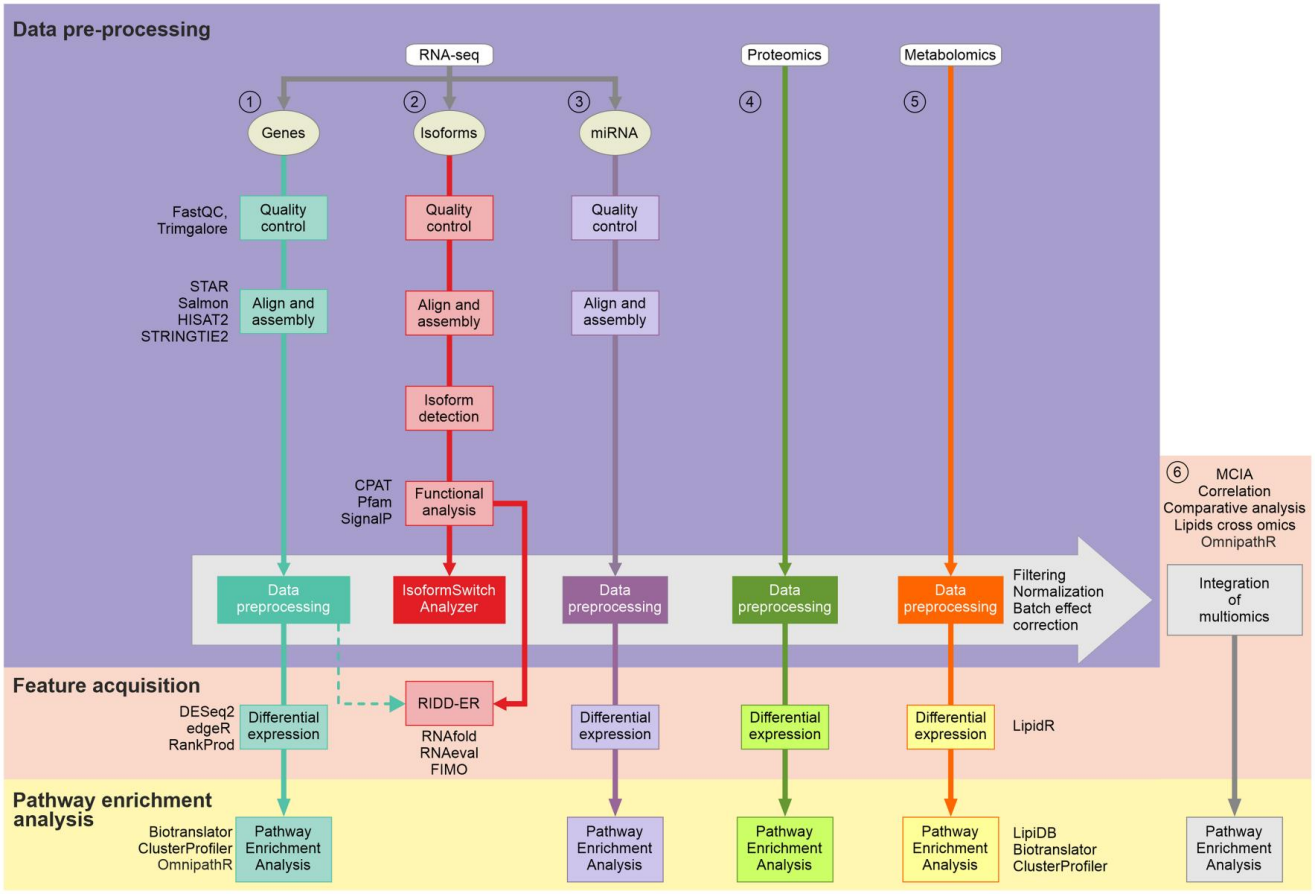
Schematic overview of MultiOmicsIntegrator (MOI). The pipeline can perform simultaneously individual and integrated analyses depending on the available data. Individual analysis can start from RAW RNA-seq data, followed by quality control, align and assembly steps, resulting in a feature matrix of genes (1), miRNAs, (2) or isoforms (3). Isoform analysis holds a separate sub-workflow in MOI. (4) Proteomic feature matrices follow similar steps to the RNA-seq analysis, adjusted with suitable algorithms. (5) Metabolomic analysis starts with a metabolomics matrix, then filtering and mstus normalization are performed and lipidr explores differentially expressed lipid classes and different expression patterns regarding saturation level or chain length. Subsequently, LipiDB associates genes with various lipids of interest and pathway enrichment analysis is performed on the associated features. If supplied with multiple omics data, MOI can integrate them (6) after the step of the corrected matrices (isoformSwitchAnalyzer step for isoforms, data preprocessing for the rest) with MCIA. Another method of integration is to explore the existence of genes associated with metabolomics available across omics, perform correlation analysis between two feature matrices, or to explore databases like OmnipathR. Furthermore, a comparative analysis of available feature signatures will indicate their semantic similarity. At the end, pathway enrichment analysis and vizualization of networks can be performed on the set of integrated features.

The first part of the MOI pipeline is dedicated to obtaining a clean abundance feature matrix (Fig. 1).

#### 2.2.1 Acquisition of abundance matrices

For RNA-seq, the workflow can start directly from SRA codes and, after performing quality control and basic quality steps (like “trimming”), it will align/assemble the reads into transcripts with tools such as salmon (Patro *et al.* 2017). These processes constitute one sub-workflow that was developed by modifying nf-core/rnaseq. Analysis of isoforms in the transcriptome is achieved by preparing the genome in a splice-aware fashion to enable quantification of the abundance of each isoform based on the read counts mapped to specific exons or junctions. To further elucidate their potential function, we include several tools that can annotate transcripts regarding their coding potential, presence of protein domains, or signal peptides (Wang *et al.* 2013, Almagro Armenteros *et al.* 2019, Mistry *et al.* 2021). Notably, these tools have a wide range of applications and can be utilized in different scenarios, e.g. functional annotation of ncRNAs.

At the end of this step, the user has an abundance matrix of genes, isoforms, miRNAs or ncRNAs depending on the reference genome provided and the specific demands of the analysis.

MOI can also be used for proteomics and metabolomics data, both of which require an abundance matrix as input. We do not include all pre-processing steps for raw mass spectrometry data because existing pipelines provide adequate output, which is not the case for RNA-seq data

#### 2.2.2 Pre-processing of abundance matrices

The final standalone module of this part of MOI holds tools for various standard data pre-processing steps. Specifically, the user can first filter their data setting specific thresholds and then normalize according to the type of data they have (with edgeR or quantile methods). Omics data are characterized by their high dimensionality that can often be confounded with batch effects. Correcting batch effects is crucial to improve the accuracy and reliability of downstream analyses, such as differential expression or pathway analysis. In MOI, this step takes place after data normalization and is performed with sva R package (Parker *et al.* 2014). The inclusion of many different methodologies can capture the different modalities of different data types.

### 2.3 Feature acquisition

The second part constitutes the acquisition of features of interest. This part can be applied to both single and multi-omics analyses. This is accomplished using both data-driven and biology-driven approaches, see details below and Fig. 1.

#### 2.3.1 Differential expression analysis

MOI provides popular algorithms used for differential expression analysis (Supplementary Table S1) in addition to non-parametric algorithms for this task (Del Carratore *et al.* 2017). All methods can effectively handle the substantial number of genes and low counts typically available in omics datasets and can accommodate a wide range of experimental set ups. In addition, a user can insert a custom design matrix, which allows extensive parameterization in conducting desired comparisons.

#### 2.3.2 Differential isoform usage

MOI includes a specialized sub-workflow that evaluates the relative abundance of gene isoforms, estimates isoform switches and indicates their potential genome-wide implications on transcriptional regulation events. To investigate isoform switching events, isoforms are grouped based on their expression patterns across conditions. Then statistical tests are performed to assess the significance of isoform switches within each group. These steps are performed with isoformSwitchAnalyzer (Vitting-Seerup and Sandelin 2019).

#### 2.3.3 Differential lipidomic analysis—LipiDB

Metabolomics analysis follows similar steps to differential expression analysis described above and additionally incorporates tailored processes for lipid data such as lipid class annotation, differential expression, and enrichment analysis of different classes of lipids, chain length, and degree of saturation of the hydrocarbon chains. For this, we leveraged the bioconductor packages like lipidr (Mohamed and Molendijk 2020). In addition, we introduced a custom tool that we named LipiDB to annotate lipid species. LipiDB associates genes with specific lipid classes existing in the data using a manually curated database and produces a heatmap of genes related to available lipid classes.

#### 2.3.4 Exploratory analysis across omics

If multiple omics are available, MOI offers various ways to integrate them. The pipeline can perform exploratory investigation of the common differentially expressed features across omics types. For example, LipiDB accepts inputs from additional omics layers and outputs a heatmap depicting common genes related to lipids found in the dataset across omics types. Furthermore, MOI can retrieve potential targets of differentially expressed miRNAs with tools like multimiR, thus enabling the user to explore their presence across omics layers.

#### 2.3.5 Correlation analysis

Apart from the exploratory analysis described above, MOI can perform correlation analysis between the differentially expressed features of diverse omics. The correlation tool is used to estimate Pearson or Spearman correlation score between two feature expression matrices of any type. This can be particularly useful in the context of ncRNA analysis. The user can correlate the differentially expressed features, retaining only those above a particular threshold for pathway enrichment analysis, an approach that will produce more robust results than an alternative strategy where all RNAs are retained for subsequent analyses.

#### 2.3.6 Integration of multi omics with Multiple Co-Inertia Analysis

Multiple Co-Inertia Analysis (MCIA) (Meng *et al.* 2014) offers a more sophisticated approach to integrate two or more data types simultaneously (Fig. 1), after the step of batch effect correction or the differential expression analysis step. MCIA, widely used in the field, is a data-driven integration method that can identify common patterns across multiple datasets, including different omics datasets.

#### 2.3.7 Semantic correlation analysis

MOI includes standalone modules that offer a high-level comparison of the data, since the requirements to apply machine learning algorithms (i.e. a higher number of samples than features) are not met in many experimental set-ups. The semantic correlation analysis tool compares the semantic profiles of gene signatures by assessing the semantic similarities of sets of Gene Ontology Biological Process terms across omics types. This method was previously developed by authors of this paper (Koutsandreas *et al.* 2016).

### 2.4 Pathway enrichment analysis

The third conceptual part of the pipeline, available for individual as well as integrated analyses, describes how to identify key molecular changes and pathways characteristic of the condition/treatment of interest (Fig. 1). MOI translates all available omics layers to the gene level to enable incorporation of all data into pathway enrichment analysis, resulting in a set of prioritized genes derived from all available omics layers.

#### 2.4.1 Biotranslator

In MOI, pathway enrichment analysis is performed with biotranslator, an algorithm developed previously by the members of our team (Koutsandreas *et al.* 2016). This tool can uncover important biological processes and prioritize genes using a unique workflow that conducts statistical and network analyses on biological hierarchical vocabularies to pinpoint and rank significantly enriched processes alongside their key hub genes. Alternative well-cited and popular choices that MOI also offers can be found in Supplementary Table S1.

#### 2.4.2 OmnipathR

OmnipathR is a tool that enables network and pathway reconstruction based on protein–protein interactions and known regulatory networks (Türei *et al.* 2016). The output consists of a network with useful information about the role of the feature in the signaling pathway (e.g. whether it is a ligand or a transcription factor), the subcellular location, the type of omics where it is found in the user’s dataset, or annotated based on specific criteria the user chooses. OmnipathR can also be used on a set of differentially expressed features, facilitating another feature selection/integration method offered by MOI.

## 3 Results—discussion

To showcase the utility of MOI, we challenged it to perform both individual and integrated analyses of different datasets. We first employed the pipeline to implement isoform analysis, using RNA-seq data from a study describing Parkinson’s disease (PD) (Dumitriu *et al.* 2016). This analysis revealed differential expression of isoforms of *SNCA* (Supplementary Fig. S1A), the gene that codes for alpha-synuclein and of known importance in PD pathology (Dumitriu *et al.* 2016), as well as previously unexplored genes, such as *GSDMD, OPA1, RIC1,* and* TERF1*. In addition, we were able to investigate genome-wide implications of isoform switching events, which revealed an increase in alternative transcript starting and ending events, as well as an increase in the length of UTR regions (Supplementary Fig. S1B and C). Next, to showcase the data-driven integration part of MOI, we used datasets from breast cancer (BC) for transcriptomics (coding and ncRNA), proteomics, and metabolomics from Cancer Cell Line Encyclopedia (CLLE) (Barretina *et al.* 2012), investigating the differences between triple negative breast cancer (TNBC) and other BC subtypes. We were able to retrieve experimentally validated targets like *XBP1* and *DGAT2* known to be implicated in TNBC pathology (Almanza *et al.* 2022), as well as biological pathways for adhesion, proliferation, and secretion, showcasing the robustness of the pipeline even when applied on a test study. More details can be found in Supplementary Figs 2–7. The set of acquired features produced by this analysis harbor potential prognostic value (Supplementary Fig. 8). These diverse case studies demonstrate the pipeline’s versatility and applicability to different research questions.

With MOI, we wanted to build a pipeline that could perform comprehensive analysis of multiple omics datasets, while at the same time maintaining a relative simplicity of use. Notably, other existing pipelines offer only fragmented solutions, focusing on some parts of the analysis (Table 1 and Supplementary File Table 3). For example, MOI facilitates, in parallel, both individual analyses and integration approaches, streamlining the comprehensive exploration of multiple omics datasets and allowing for a seamless comparison of results from the two different analytical perspectives. In addition, it holds a variety of tools not available in other pipelines (see Supplementary Table 3).

**Table 1.** Comparison of utilities offered by MOI with other existing pipelines.

	MOI	nf-core/rnaseq	nf-core/rnasplice	nf-core/quantms	nf-core/metaboigniter	Galaxy-P
Preprocessing steps	*	*	*	*	*	*
Functional annotation of RNA	*					Partially
Differential isoform usage	*		*			
Differential feature expression	*	*	*			*
Differential lipidomic expression	*					
Integration of multiple omics (data-driven)	*					
Integration of multiple omics (biology-driven)	*					
Pathway Enrichment Analysis	*					Partially
Semantic correlation analysis	*					

The pipeline’s modular design renders it highly flexible and dynamic, allowing users to easily select and customize individual modules to meet specific research needs. Furthermore, MOI was developed using nextflow and Docker, allowing it to be easily deployed and scaled to accommodate large datasets and high-throughput analyses. Another advantage of this architecture is that it allows for straightforward combination and extension. We propose that several sub-workflows could be adopted by the nf-core community for use in various scenarios, such as integrating with workflows dedicated to other omics data types. Specifically, key elements of MOI, particularly its integration capabilities, can be applied to analyze other omics types. For instance, abundance matrices from any omic type can be incorporated during data pre-processing or at the MCIA step. Furthermore, if additional omics data are mapped to the gene level, MOI can integrate them using tools for exploratory multi-omics analysis, correlation analysis, or semantic (comparative) analysis, as well leveraging tools such as multiMiR, lipidDB, biotranslator, or OmnipathR for more high level integration.

Users with basic coding expertise can easily use the MOI pipeline, as they will only need to modify one parameter file, following the detailed documentation available online. To ensure ease of use, we have provided comprehensive documentation on our GitHub page, which thoroughly explains how to execute each of the workflows. This includes detailed documentation of each workflow, example outputs for the major tools and workflows as well as several example YAML files, where users need only adjust clearly marked input and output parameters. Additionally, we have made the documentation available in a Read-The-Docs format for greater accessibility. All layers of code are freely accessible, which could potentially benefit other researchers in the field. We hope that MOI will be a valuable resource for researchers working with omics data.

## Supplementary Material

vbae175_Supplementary_Data

## Data Availability

Supplementary figures and tables are available in the Supplementary File. The pipeline is hosted at: MOI. Extensive Documentation can be found at: ReadTheDocs documentation.
