# Supplementary material for: MultiOmicsIntegrator: a nextflow pipeline for integrated omics analyses
Source: Bioinform Adv. 2024 Nov 14;4(1):vbae175. doi: 10.1093/bioadv/vbae175 (PMC11576358; doi:10.1093/bioadv/vbae175)
Supplement: vbae175_Supplementary_Data [file vbae175_supplementary_data.docx]

**Supplementary File**

MultiOmicsIntegrator – MOI: A pipeline for integrated omics analyses


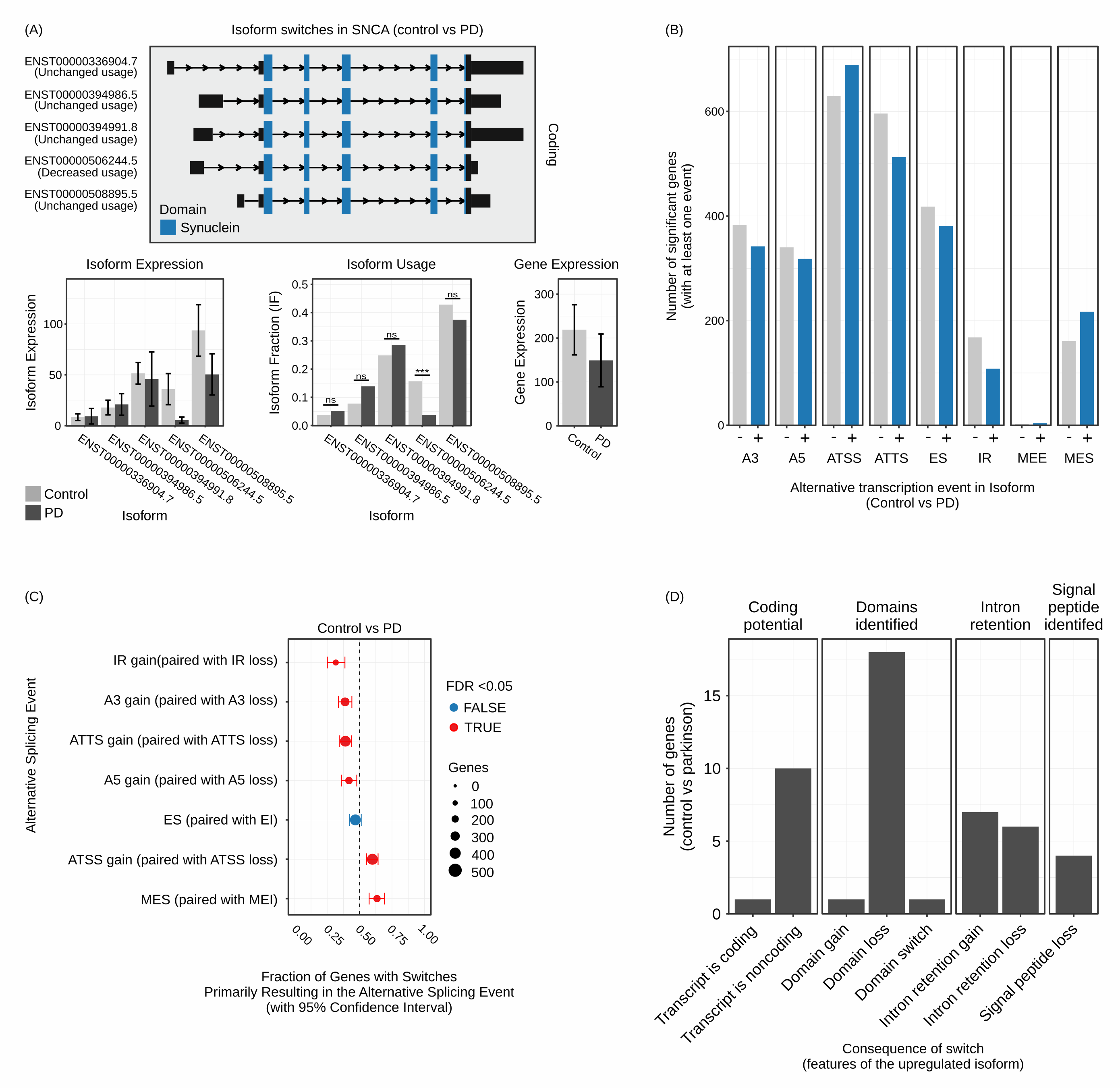


**Supplementary Figure 1: Demonstrational data of isoform analysis on Parkinson’s disease publicly available data.** (A) Different isoforms of SNCA mRNA are detected and annotated with respect to their coding potential and protein domains. Fold changes on the expression and their relative usage in Parkinson’s disease (PD) samples is displayed. Additionally fold changes at the level of the whole gene expression are shown. Plots shown in supplementary Figure 1B highlight isoform switching occurrences, which are detailed in supplementary Table 2. Genome-wide enrichment of isoform switches entails tallying switches of a particular type and contrasting gains versus losses using standard statistical methods. As such, boxplots in supplementary Figure 1B depict the number of genes encompassing functional impacts of isoform switching. Splicing events are depicted on the X axis and are as follows: A3 and A5 refers to 3’ and 5‘untranslated regions, respectively; ATSS and ATTS are alternative transcript starting and termination sites, respectively; ES is exon skipping and IR is intron retention; MEE and MES refer to mutually excluded exons and mutually excluded splicing sites, respectively. In our dataset we can see that we have gain in ATSS in PD samples. In supplementary Figure 1C we can explore which of the isoform events is enriched over the others, in other words we can see the comparison of enrichments. In our case we can see that only Exon Switching did not pass the significance level and all other isoform switching events were enriched between conditions. Supplementary Figure 1D illustrates the implications of the isoform switches at a functional level, i.e., the consequences of isoform switches regarding coding potential, intron retention, and loss or gain of protein or signal domains.


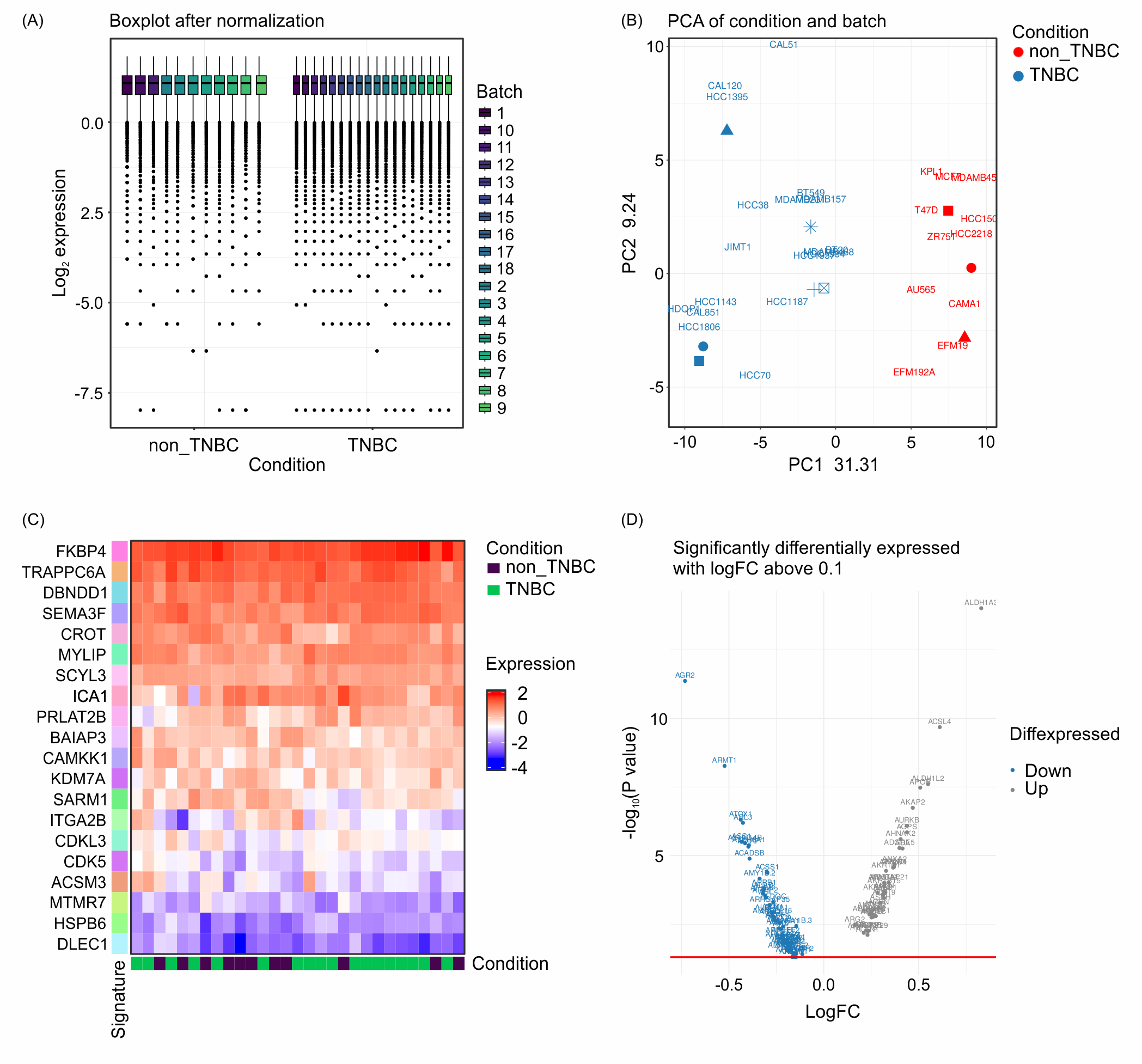


**Supplementary Figure 2: Demonstrational outputs from individual analyses of RNA-seq data.** We performed pairwise comparisons of triple negative breast cancer (TNBC) subtype versus non TNBC using data on breast cancer cell lines from the CCLE data portal. (A) Box plots of samples after filtering and normalizations steps for conditions TNBC and non TNBC. (B) PCA of cleaned data for batch effect samples provide quality control of the data. Heatmaps (C) and Volcano plots (D) offer visual indications of differentially expressed features.


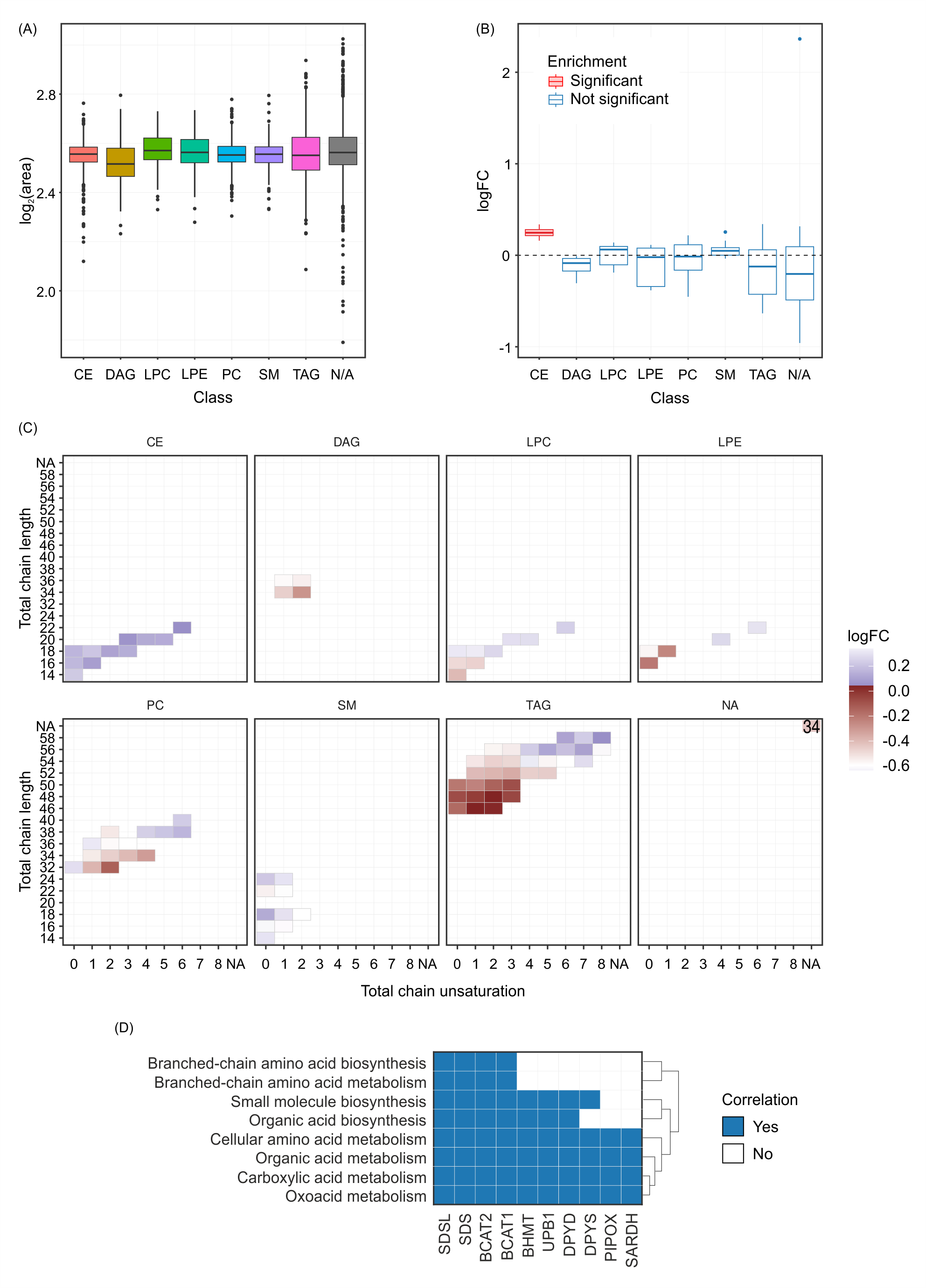


**Supplementary Figure 3: Demonstrational outputs from individual analyses of metabolomic data**. We performed pairwise comparisons of TNBC subtype versus non TNBC using data on breast cancer cells lines from the CCLE data portal. Box plots of the relative fold change for each class of lipids is displayed (A) along with the output showing whether that fold change is enriched between conditions (B). The different classes of lipids shown are ceramides (CE), diglycerides (DAG), lysophosphatidylcholines (LPC), lysophosphatidylethanolamines (LPE), phosphatidylcholine (PC), sphingomyelin (SM) and triaglycerides (TAG). Distribution of saturation levels for each class of lipids is shown in (C) and the most enriched pathways found using Biotranslator in which these lipids participate is shown in (D).


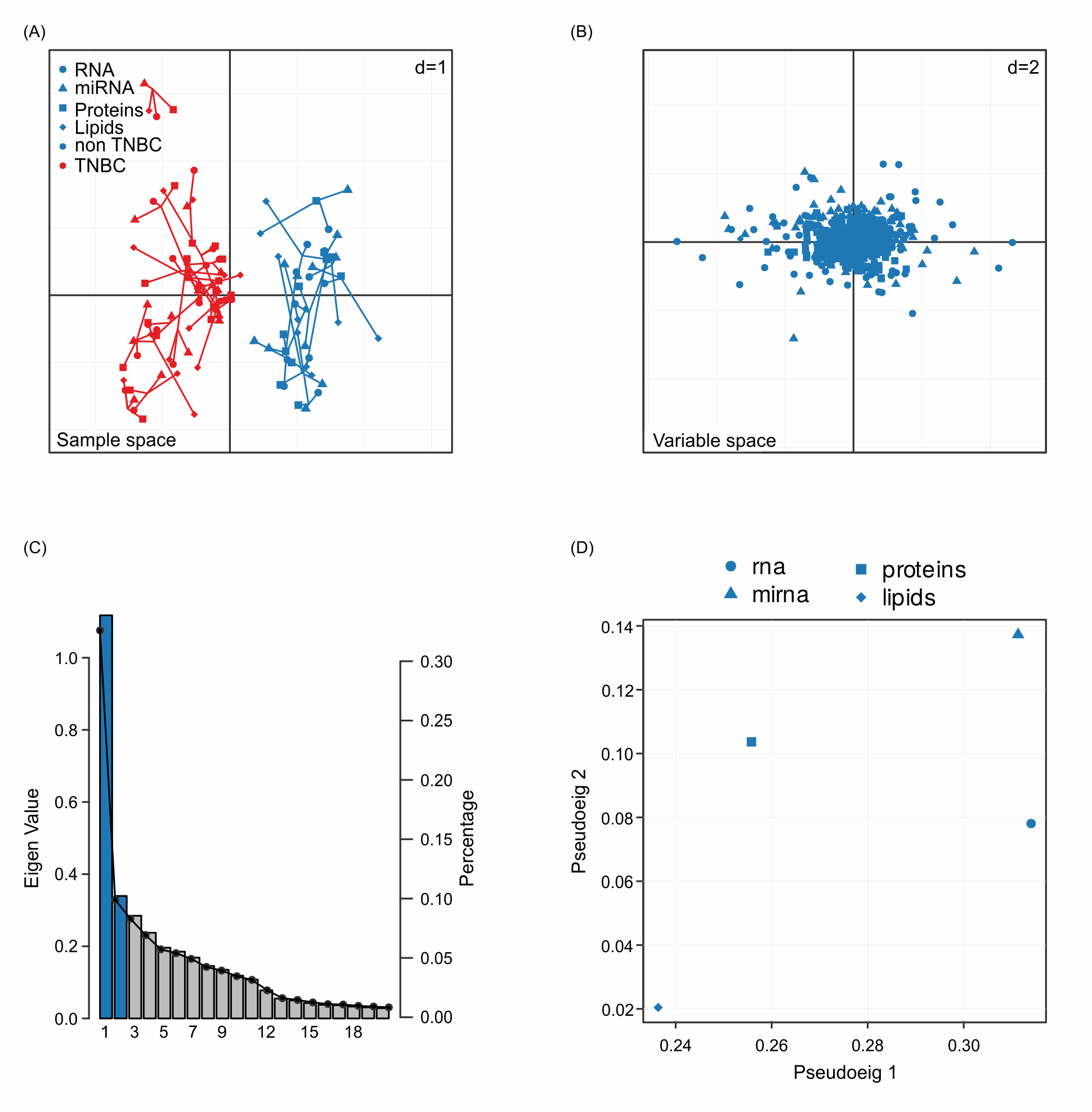


**Supplementary Figure 4: Demonstrational data from integration analysis.** The integration analysis was performed using the same datasets that were used for Figures 2 and 3. (A) MCIA reports the PCA of the sample space where we can see how samples differentiate according to the phenotype of interest. (B) In addition, variables are projected on the same space to explore the relative contribution of each variable to the distinction of the phenotypes. Elbow plots (C) inform us about the significant principal components and in panel (D) the space of the pseudo-eigen values of the different datasets is displayed, as an indication of the relative contribution to the variance observed.


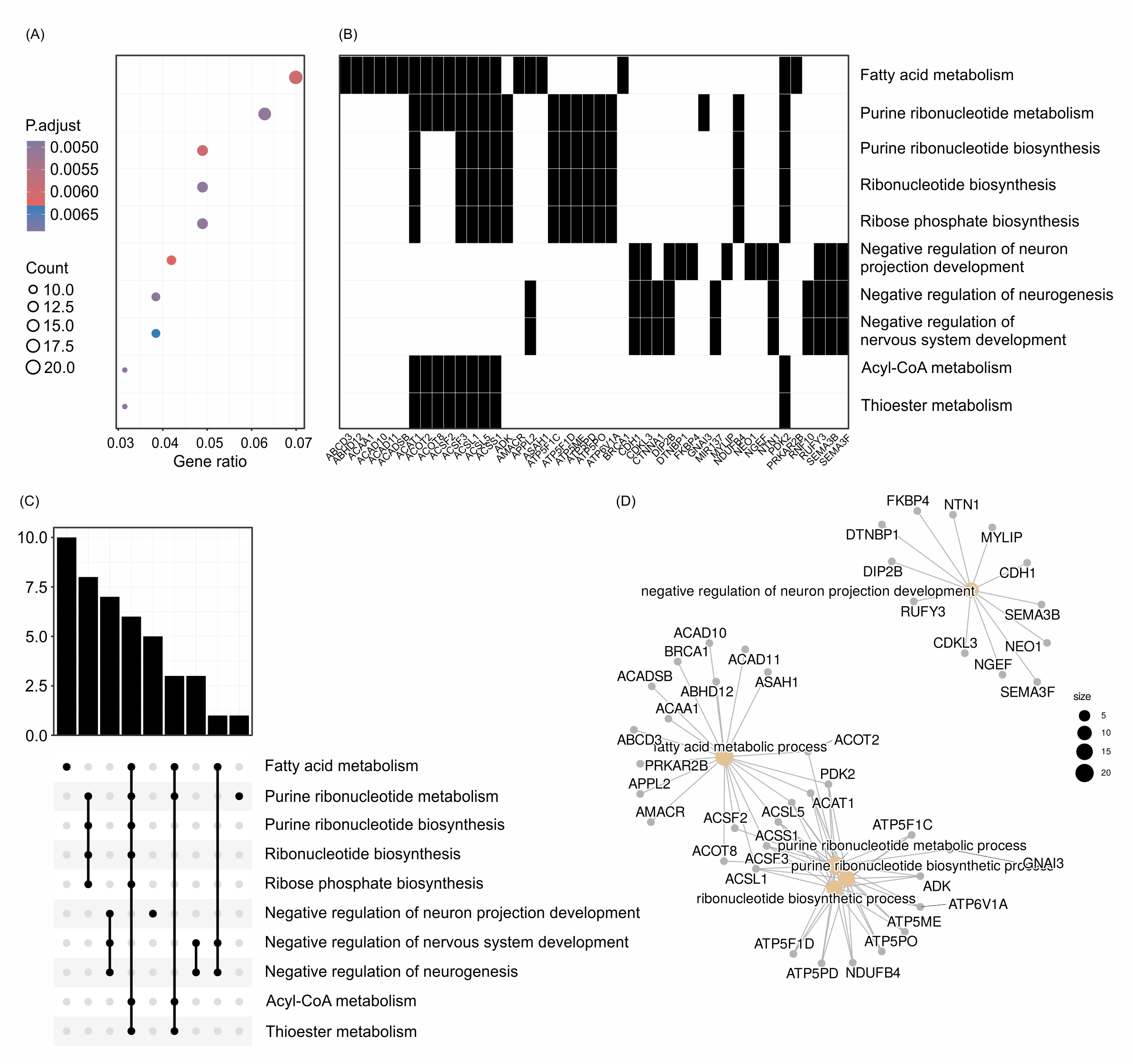
**Suplementary Figure 5: Demonstrational data from functional analysis performed with clusterProfiler.** ClusterProfiler can be utilized by individual analyses or after the integration step of MCIA. Outputs include heatmaps of enriched processes (A) and the top features that participate in these processes (B), as well as tree plots of significant pathways (C) and the network that these pathways form (D).


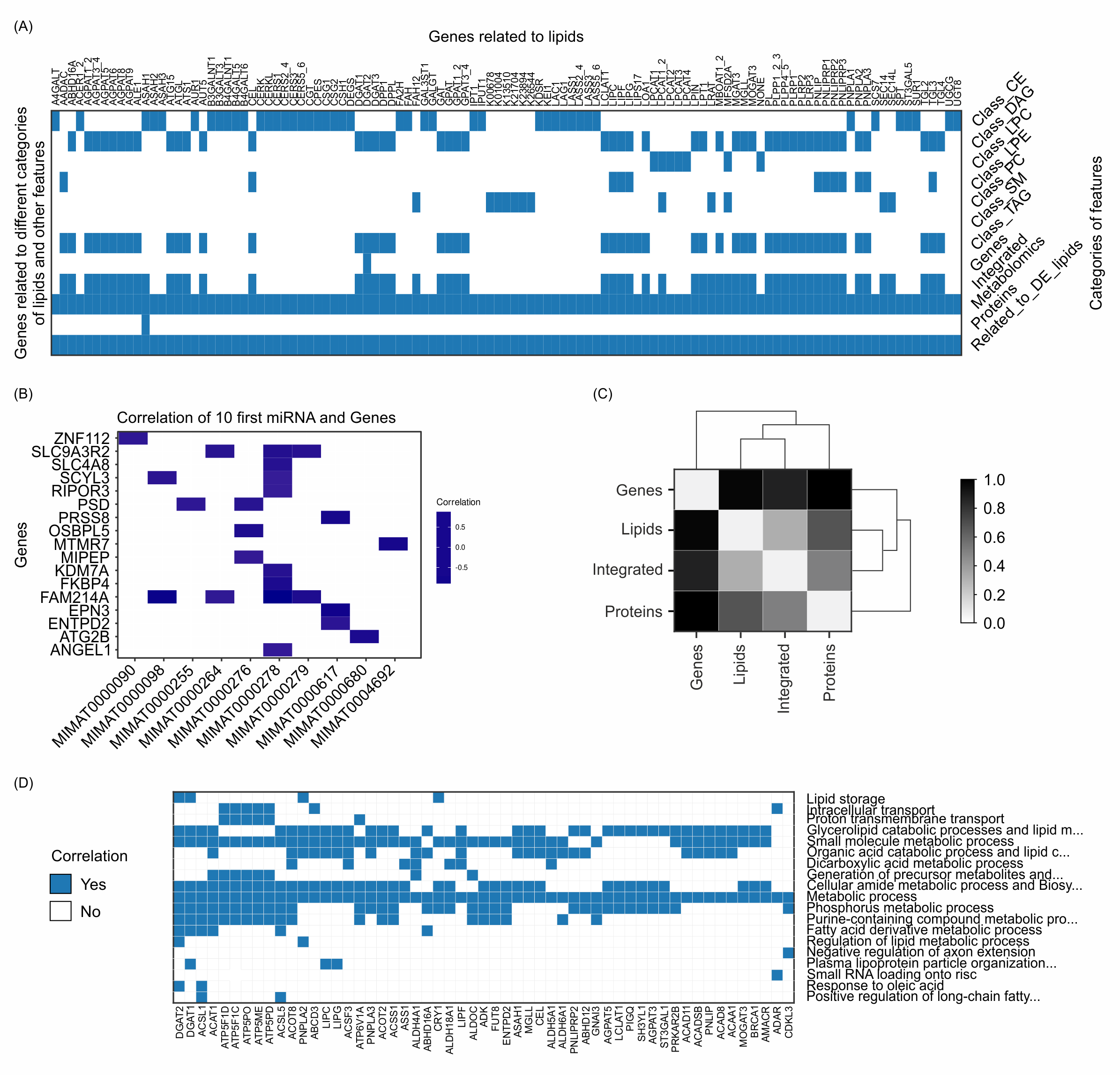


**Supplementary Figure 6: Additional integration methods offered by MOI**. (A) MOI has unique tools for associating genes to the molecules found in metabolomic datasets and visualizing them across functional lipid categories and available omics data. (B) Correlation analysis performed with Pearson or Spearman can filter illuminating connection and patterns and aiding the noise mitigation for downstream analyses like pathway enrichment analysis (functional analysis). The example provided here shows the top 10 differentially expressed miRNA and their correlated genes. (C) The semantic distance matrix of feature signatures performed by our comparative analysis tool explores how closely connected regarding the biological ontologies are two signatures. (D) shows the biological processes that hold the most descriptive information of the observed phenotype, interconnected with hub genes (x axis), performed with Biotranslator.


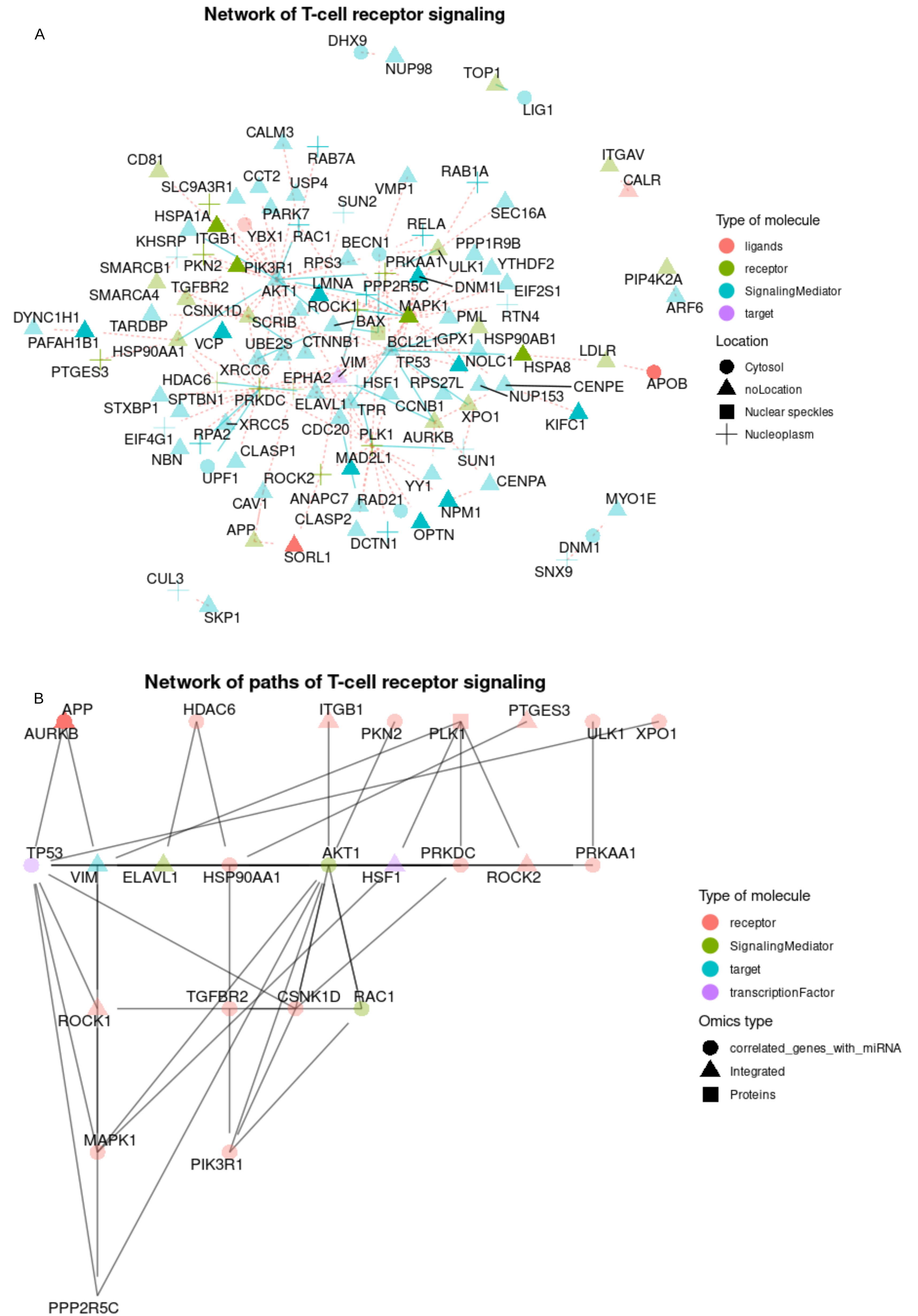


**Supplementary Figure 7: Demonstrational data from omnipathr.** Visualization of networks of prioritized features. (A) The user can use omnipathr to annotate their network based on various attributes like location (shape), type of molecule (color) and whether or not it belongs to a specific function of interest (Tcell receptor signaling linked with transparency levels). (B) Specific deregulated pathways of the network are shown. The user can also choose to annotate based on the omics of origin of the prioritized features.



**Supplementary Figure 8:** Kaplan Meier survival plot for TCGA Breast Cancer Patients. We took features characteristic of TNBC pathology originating from our integration strategy of transcriptomics, proteomics and metabolomics data of CCLE BRCA cell lines. Then we scored TCGA patients according to their relative expression of these features, forming three groups: High, Mid and Low. Then we performed a survival analysis and observed that low score on this set of features correlates with worse prognosis.

**Supplementary Table 1: Description of the most important tools used in the MOI pipeline**

| **Omics** | **Functionality** | **Tools** | **DOIs** |
| --- | --- | --- | --- |
| Genes, miRNA, isoforms | SRA download | SRA toolkit | https://doi.org/10.1093/nar/gkq1019 |
| Genes, miRNA, isoforms | Quality control | FastQC, trimgalore | https://doi.org/10.1111/gtc.12870 |
| Genes, miRNA, isoforms | Align and Assembly | Salmon, samtools, STAR, Hisat2, StringTie2, | <https://doi.org/10.1101/021592>, <https://doi.org/10.1093/gigascience/giab008>, <https://doi.org/10.1093/bioinformatics/bts635>, 10.1088/1742-6596/2179/1/012038,  <https://doi.org/10.1186/s13059-019-1910-1> |
| Genes, miRNA, isoforms, proteins, lipids | Data preprocessing | R packages: edger, limma, sva, ggplot2, ComplexHeatmap | https://doi.org/10.1093/bioinformatics/btp616, <https://doi.org/10.1093/nar/gkv007>, <https://doi.org/10.1093/bioinformatics/bts034>, <https://doi.org/10.1002/wics.147>, https://doi.org/10.1002/imt2.43 |
| Proteins, lipids | Specific for proteins and lipids | R packages: preprocesscore, mstus normalization | git_url <https://git.bioconductor.org/packages/preprocessCore>, |
| Lipids | Specific for lipids | R packages: lipidr | 10.1021/acs.jproteome.0c00082 |
| Genes, miRNA, isoforms, proteins, lipids | Differential expression analysis | R packages: DESeq2, edger, RankProd, ggplot2 ComplexHeatmap | <https://doi.org/10.1186/s13059-014-0550-8>, https://doi.org/10.1093/bioinformatics/btp616,  10.1093/bioinformatics/btx292, https://doi.org/10.1002/wics.147, https://doi.org/10.1002/imt2.43 |
| Genes, miRNA, isoforms, proteins, lipids | Correlation analysis | R package stats | - |
| Genes, miRNA, isoforms, proteins, lipids | Pathway enrichment analysis | Clusterprofiler, Biotranslator, OmnipathR | <https://doi.org/10.1089/omi.2011.0118>, 10.4018/IJMSTR.2016040103, 10.15252/msb.20209923 |
| Lipids | Specific for lipids pathway enrichment analysis | Custom tool: Lipidb | - |
| Genes, miRNA, isoforms, proteins | RIDDER (module to identify IRE1 substrates) | gRIDD, RNAeval, fimo | <https://doi.org/10.1038/s41467-021-27597-7>, https://doi.org/10.1093/nar/gkn188 |
| Genes, miRNA, isoforms | Functional annotation | CPAT, signalP, pfam | <https://doi.org/10.1093/nar/gkt006>, <https://doi.org/10.1038/s41587-021-01156-3>, https://doi.org/10.1093/nar/gkaa913 |
| Genes, miRNA, isoforms, proteins | Secondary structure prediction | RNAfold, RNAeval | https://doi.org/10.1093/nar/gkn188 |
| Genes, miRNA, isoforms, proteins | Find motif | fimo | https://doi.org/10.1093/nar/gkn188 |
| Isoforms | Genome wide isoform analysis | IsoformSwitchAnalyzer | https://doi.org/10.1093/bioinformatics/btz247 |

**Supplementary Table 2: Abbreviations of Isoform Switching Events**

| **Abbreviation** | **Classification** |
| --- | --- |
| ES | Exon Skipping |
| MES | Multiple Exon Skipping |
| MEE | Mutually Exclusive Exons |
| IR | Intron Retention |
| A5 | Alternative 5’ splice site |
| A3 | Alternative 3’ splice site |
| ATSS | Alternative Transcription Start Site |
| ATTS | Alternative Transcription Termination Site |

**Supplementary Table 3: Additional comparisons with existing pipelines regarding available tools**

|  | MOI | nfcore/rnaseq | Nfcore/ rnasplice | nfcore/quantms | nfcore/metaboigniter | Galaxy-P |
| --- | --- | --- | --- | --- | --- | --- |
| sva, svaseq, ComBat | Y |  |  |  |  |  |
| EdgeR,  DESeq  DESeq2  RankProd | Y | DESeq2  edgeR | edgeR |  |  | EdgeR  DESeq2 |
| SignalP, Pfam search and cpat | Y |  | Y |  |  | Y |
| Rnafold, gridd | Y | Y |  | Y |  | RNAfold |
| LipidR lipiDB | Y |  |  |  |  |  |
| MCIA | Y |  |  |  |  |  |
| MultimiR, omnipathR | Y |  |  |  |  |  |
| Biotranslator and omipathR | Y |  |  |  |  |  |
| Comparative analysistool | Y |  |  |  |  |  |
